# Sexuality among fathers of newborns in Jamaica

**DOI:** 10.1186/s12884-015-0475-6

**Published:** 2015-02-21

**Authors:** Peter B Gray, Jody-Ann Reece, Charlene Coore-Desai, Twana Dinnall-Johnson, Sydonnie Pellington, Maureen Samms-Vaughan

**Affiliations:** Department of Anthropology, University of Nevada, 4505 S. Maryland Parkway, Box 455003, Las Vegas, NV 89154-5003 USA; Department of Child and Adolescent Health, University of the West Indies, Mona Campus, Mona, Jamaica

**Keywords:** Fatherhood, Men, Parents, Caribbean, Sexual function, Sexual relationship

## Abstract

**Background:**

While a growing body of research has addressed pregnancy and postpartum impacts on female sexuality, relatively little work has been focused upon men. A few studies suggest that a fraction of men report decreases in libido during a partner’s pregnancy and/or postpartum, with alterations in men’s sexual behavior also commonly aligning with those of a partner. Here, we investigate sexuality among fathers of newborn children in Jamaica. In Jamaica, as elsewhere in the Caribbean, relationship dynamics can be fluid, contributing to variable paternal roles and care, as well as a high fraction of children born into visiting relationships in which parents live apart from each other.

**Methods:**

During July-September, 2011, 3410 fathers of newborns with an average age of 31 (SD = 8) years participated in the fatherhood arm of a national birth cohort study (JAKids). These fathers answered questions about sociodemographic background, relationship quality and sexuality (e.g., various components of sexual function such as sex drive and sexual satisfaction as well as number of sexual partners the previous 12 months and sexual intercourse the previous week) during a visit to a hospital or birth center within a day or two of their child being born.

**Results:**

Showed that sex drive was more variable than other components (erections, ejaculation, problem assessment) of sexual function, though sexual satisfaction was generally high. Thirty percent of men reported two or more sexual partners the previous 12 months. Nearly half of men indicated not engaging in sexual intercourse the past week. Multivariate analyses showed that relationship status was related to various aspects of men’s sexuality, such as men in visiting relationships reporting more sexual partners and more openness to casual sex. Relationship quality was the most consistent predictor of men’s sexuality, with men in higher quality relationships reporting higher sexual satisfaction, fewer sexual partners, and higher frequency of sex, among other findings.

**Conclusions:**

These results provide an unusually large, quantitative look at men’s sexuality during the transition to fatherhood in Jamaica, offering helpful insight to would-be parents, clinicians or others seeking to anticipate the effects of a partner’s pregnancy on men’s sexuality.

## Background

A small but growing body of research has addressed the influence of pregnancy and postpartum phases on sexuality [1,2]. An understanding of these effects is important for various reasons, including to counsel would-be parents on what can be dramatic yet normal alterations in partners’ sexual dynamics. The vast amount of this literature investigating the effects of having a child on sexuality has focused on women, however. Decreases in women’s sexual desire and sexual frequency during late pregnancy and postpartum have been well-documented [3,4], along with moderating constitutional and contextual factors such as mode of delivery (e.g., C-section vs. vaginal birth), relationship quality (often more frequent sexual activity in higher-quality relationships), and acceptance of alternate sexual partners (e.g., post-partum sexual taboos and polygyny) [5]. But fathers also warrant attention to determine how features of their sexuality such as sexual desire, sexual behavior, and sexual satisfaction are impacted during these same transitions.

A review of 59 studies, largely drawing upon European and North American samples, noted that few studies on fatherhood and peripartum sexuality had been published [2]. At that time, the limited available data suggested that a fraction of fathers reported decreases in sexual desire during pregnancy and up to a year postpartum, along with alterations in sexual behavior during these times, such as reduced coital frequency. A handful of studies published since then have offered additional insights into fatherhood and sexuality. A longitudinal study of 30 German couples found that men’s masturbation rates were relatively constant across pregnancy and six months postpartum, although various partnered sexual behaviors such as intercourse and oral sex declined to low levels after the birth of a child [6]. In Nakic Rados et al’s [7] study of 105 Croatian men, researchers found that nearly a third of male participants reported lower sexual desire along with decreases in sexual behavior during their partner’s final trimester of pregnancy. Although men’s reported sexual satisfaction was generally high, it decreased in nearly half of men, and was highly related to relationship quality [7]. A sample of 279 men from southeastern Nigeria observed that approximately 40% reported decreased libido, over 70% reported decreased sexual behavior, and yet 28% reported engaging in extramarital sexual behavior during a partner’s pregnancy, although extramarital sexual behavior was less common among older men [8]. A small, qualitative study of Swedish fathers discussing their sexuality 3–6 months postpartum highlighted the need to renegotiate sexuality within a relationship and focus upon an infant [9]. A larger qualitative study of 128 Australian fathers drew upon questionnaire responses 6 weeks postpartum, and featured decreases in sexual behavior, and adjustments to the birth, a partner’s attention to their infant, and being tired [10].

In the present study, we build upon this limited literature to focus upon the sexuality of fathers of newborns in Jamaica. Consistent with insights from this small body of existing work, we seek to measure sexual behaviors such as intercourse, but also other facets of sexuality such as sexual desire and sexual satisfaction. We also anticipate that relationship dynamics (e.g., relationship quality and availability of alternative partners) are important elements in contextualizing men’s sexuality during this process. Since late pregnancy and early postpartum phases have the largest impact on women’s and, it seems, their partnered men’s sexuality, a focus upon fathers of newborns highlights a time when men’s sexual desire, satisfaction and behavior are apt to be in flux, given the concern over a new child, but also alterations in sleep, relationship quality, and other domains [11,12]. The Nigerian findings also recognize the importance of placing men’s sexuality in cultural context, given that the percentage of fathers in that sample reporting extramarital sex (28%) during a partner’s pregnancy is higher than indicated by European, North American or Australian findings (see [13,14] for related discussion).

### Jamaican cultural context

Family dynamics in the Caribbean, including Jamaica, have long drawn attention from scholars, and have been noted for standing out in cross-cultural perspective. Early contributions like Edith Clarke’s [15] “My Mother who Fathered Me” and Smith’s [16] “West Indian Family Structure” recognized a high prevalence of matrifocal household structures in the Caribbean, in which mothers anchor the family unit (see also [17-22]). The fluidity of sexual relationships can lead to males playing variable paternal roles, and placing heightened importance on maternal grandmothers as sources of family support. Births frequently take place within so-called visiting relationships, in which a man living separately maintains an ongoing social and sexual relationship with his child’s mother [20]. Visiting relationships sometimes transform into common-law unions, and these into formal marriage, but many also dissolve. Seventy percent of births in the Caribbean occur outside of marriage, a higher percentage than any other region of the world [20]. Households of mixed parentage often occur, meaning that fathers frequently play both biological and stepfather roles. The primary paternal responsibility of African-Caribbean men tends to be viewed as economic—as financial providers, although in practice economic constraints often mean men fail to meet this overriding standard [23]. Approximately 93% of Jamaica’s population claims African Caribbean descent, with the remainder a mixture of European, Native Caribbean (e.g., Taino), South Indian, Chinese, and Southwest Asian ancestry.

In Jamaica, recent data reveal that 49% of children are born into visiting unions, 36% common-law unions, and 15% married unions [24]. Religious affiliation and socioeconomic status are both positively associated with couples marrying, and particularly for children being born within households of already-married couples. The quality of a man’s relationship to a coresidential mate also helps predict his paternal involvement. This Jamaican background thus recognizes considerable variation in relationship and paternal dynamics, and also points toward investigating the effects of socioeconomic status on fathers’ sexuality. We draw upon these wider Caribbean and Jamaican contextual considerations to embed the present study on fathers’ sexuality with respect to relationship status (visiting/common-law/marital unions), relationship quality, expectations concerning mating fluidity, and assessments of men’s socioeconomic status. Put another way, the aims of the present study are to 1) provide descriptive quantitative data on the sexuality of Jamaican fathers of newborns, and to 2) test the potential impacts of relationship status, relationship quality, and men’s socioeconomic status on those men’s sexuality. The rationale for investigating these predictor variables is that other research suggests these variables structure features of men’s sexuality, and they are central features of the Jamaican cultural context in which this study is situated.

## Methods

### Study design and recruitment

The proposed research builds on a new, national birth cohort study (JAKids) initiated in Jamaica in summer 2011 by Samms-Vaughan, a Jamaican pediatrician and researcher. The JA Kids study included all births occurring throughout the island of Jamaica during the three month period July-September 2011. Of the 11,124 births across the island, some 9700 mothers (87% of the population) were recruited, answering questions concerning maternal behavior, relationship dynamics, and other domains at the time of their children’s birth. The fatherhood arm of the cohort study relied upon biological fathers participating by answering a standardized set of questions during the day or two postpartum when they attended birthing facilities. Responses to questions provided basic sociodemographic information as well as outcomes on various facets of men’s lives, including relationship dynamics and sexuality. Birthing centers included private centers, large public hospitals, and smaller public facilities throughout the island. These fathers were drawn from across the island with the support of an extensive team of research assistants and staff at both public and private birthing locations.

A large sample of 3410 fathers participated, which represents 31% of all Jamaican fathers who had a child during summer 2011. This sample includes both first-time and experienced fathers. Fathers were not paid for their participation in a standardized face-to-face interview with trained staff that lasted 30 minutes on average, and that took place during the one or two hours of visiting time at a birth center in the day or two after their child was born. Fathers at virtually all Jamaican birthing centers are not allowed to attend births due to space and privacy concerns, proscribing interviews during a waiting period at a birthing center. Under normal circumstances, mothers and infants return home within two days postpartum. Many fathers are unable to visit birthing centers within this time due to work or other constraints. The sample of fathers may represent a slightly more invested set of fathers overall, but can also be seen in light of the recruitment context. Some men did not answer all items. The question most omitted referred to sexual behavior the previous 7 days, completed by 2680 participants. For all other sexuality measures, number of responses ranged from 3260–3398, and for dependent variables the number of responses ranged from 3282–3390.

Ethical approval for the study was provided by the University of the West Indies/University Hospital of the West Indies Ethical Committee and University of Nevada, Las Vegas Institutional Review Board. Written informed consent was obtained from participants.

### Structured interviews

Basic sociodemographic information included items asking about men’s age and occupation. Educational attainment was scored as 1 (primary or less); 2 (junior high); 3 (some or all secondary school, as well as vocational school); or 4 (some or all tertiary school). A measure of wealth was indexed as the total number of a possible 10 household items (refrigerator, living room set, washing machine, vehicle, VCR/DVD, computer, AC, generator, fans, water tank) present and working in a participant’s home. Relationship status was divided into three categories: visiting relationship, common-law (living together), and married. Relationship quality was measured by a 17-item scale drawn from the Avon Longitudinal Study of Pregnancy and Childbirth (ALSPAC: see [25]) based on questions such as “Did you think your relationship with your baby’s mother would end soon?”), with all questions referring to relationship quality “before the pregnancy”. Answers ranged from “almost always” to “almost never”, and possible scores on this measure of relationship quality were from 17 (highest quality) to 68 (lowest quality). Sexual function was assessed by administration of the “Brief Male Sexual Inventory” [26], which distinguishes four components of sexual function (sex drive, erections, ejaculation, and problem assessment) and also quantifies sexual satisfaction over the preceding 30 days. Men’s sexuality was also assessed by three individual items (“With how many different partners have you had sex with in the past 12 months?”; “I can imagine myself being comfortable and enjoying “casual” sex with different partners?”; and “How many times have you had sexual intercourse during the past 7 days?”). The item about “casual” sex was scored from strongly disagree (1) to strongly agree (9).

### Statistical methods

Descriptive data are reported as frequencies and means (SD). Univariate and multivariate models rely upon general linear models. Multivariate models enabled testing for predictive ability of relationship status, relationship quality, age, and male socioeconomic status (education and wealth) on components of sexual function, sexual satisfaction and number of sexual partners. Alpha was set to 0.05, and two-tailed tests were employed.

## Results

### Descriptive sociodemographic and sexuality data

A sample of 3410 fathers of newborns participated. Men’s ages ranged from 16–69, with a mean of 30.6 years of age (SD = 8.0 years) and median of 29 years of age. With respect to educational attainment, 7% completed primary school or less, 31% had some or completed lower secondary (“Jr. High”), 51% had some or completed secondary school, and 11% had some or completed tertiary school. The most common occupations were farming (6.5%), followed by masonry (5.3%), self-employed (3.9%), construction (3.7%), security (3.1%), electrician (3.1%), taxi driver (3.0%), carpenter (2.5%), welder (2.3%) and police officer (2.3%). Seventy-six percent of fathers indicated being the primary wage earner. With respect to wealth, 12% had 8–10 of the 10 material items, 46% had 5–7 items, 27% had 3–4 items, and 14% had 0–2 items. Sixteen percent of fathers reported being married, 47% in common-law unions, and 33% in visiting relationships, with 4% indicating other/not stated. Relationship quality scores ranged from 17 (all answers of “almost always”: higher quality) to 68 (all answers of “almost never”: lower quality), with 30% of fathers scoring 17–20, 22% scoring 21–24, 13% scoring 25–28 and 4% scoring 40 and above.

Descriptive data for sexuality measures are given in Table 1. These data show that variation in men’s sex drive is more pronounced than variation in men’s erections, ejaculations or problem assessments. Sexual satisfaction is generally high, with 87% of men reporting being mostly or very sexually satisfied. Thirty percent of fathers of newborns reported two or more sexual partners the past 12 months, and a small fraction indicate being quite open to casual sex. Nearly half of all men reported no sex the previous week, as might not be surprising given that these are fathers of newborns.

**Table 1:** **Descriptive sexual function and behavioral measures for Jamaican fathers of newborns**

**Sexuality variable**		**Percentage of fathers**
**Sex drive (past 30 days)**		
	0-4 (lower sex drive)	29%
	5-6	37%
	7-8 (higher sex drive)	35%
**Erections (past 30 days)**		
	0-6 (lower function)	9%
	7-9	18%
	10-12 (higher function)	73%
**Ejaculations (past 30 days)**		
	0-4 (lower function)	5%
	5-6	4%
	7-8 (higher function)	91%
**Problem Assessment (past 30 days)**		
	0-6 (more problems)	4%
	7-9	6%
	10-12 (fewer problems)	90%
**Overall, during the past 30 days, how satisfied have you been with your sex life?**		
	Very or mostly dissatisfied	3%
	Neutral or mixed	10%
	Mostly satisfied	17%
	Very satisfied	70%
**With how many different partners have you had sex with in the past 12 months?**		
	1	71%
	2	13%
	3	6%
	4	4%
	5+	7%
**I can imagine myself being comfortable and enjoying “casual” sex with different partners.**		
	1-3 (largely disagreeing)	73%
	4-6 (neutral)	17%
	7-9 (largely agreeing)	10%
**How many times have you had sexual intercourse during the past 7 days?**		
	No response	24%
	0	42%
	1	14%
	2	9%
	3	6%
	4+	6%

### Relations between sociodemographic factors and sexuality data

**Figure 1:**
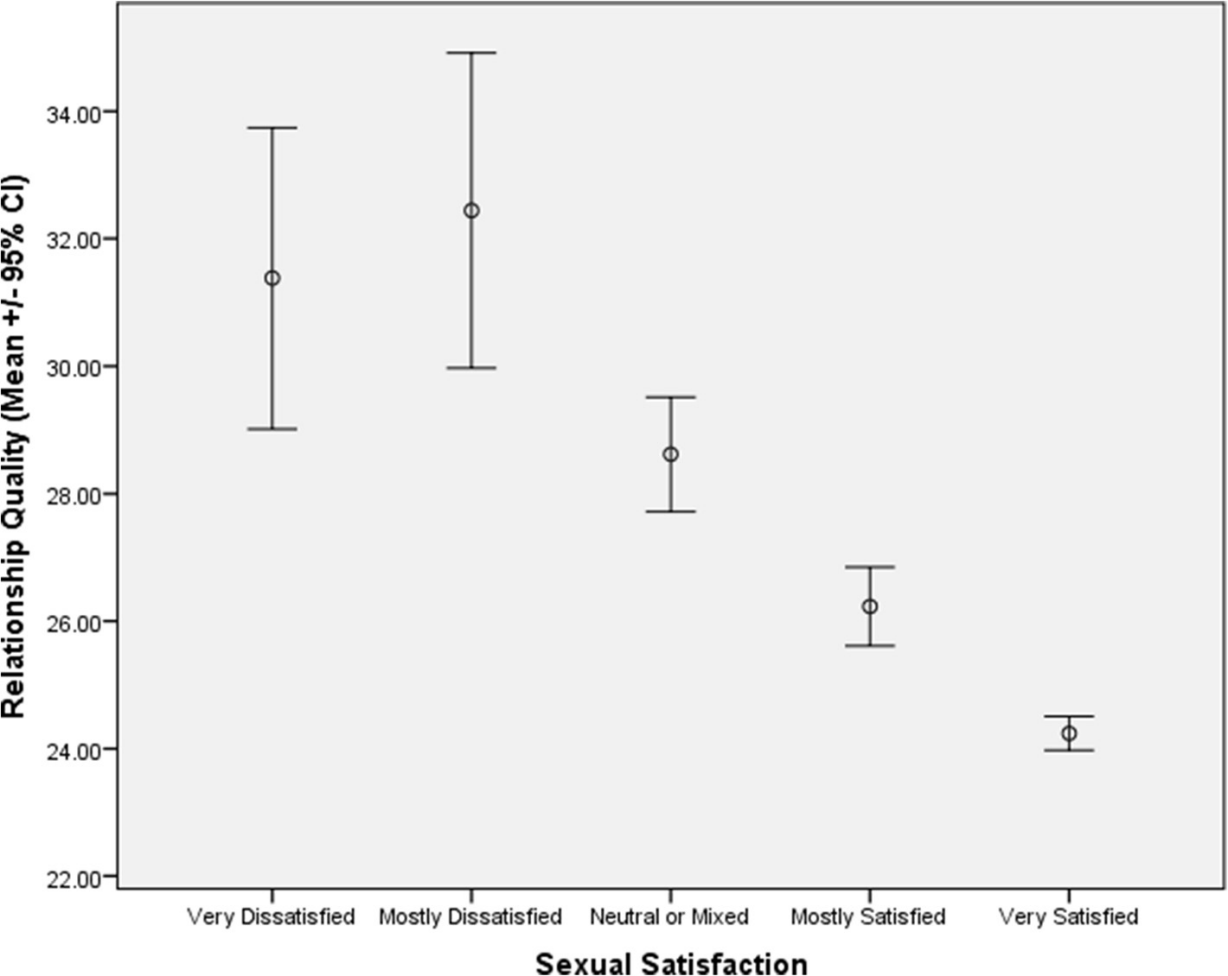
**Relationship quality is inversely associated with sexual satisfaction.** Men reporting more sexual satisfaction show higher relationship quality (lower relationship quality scores indicate higher quality).

Univariate Pearson correlations among sexuality variables, relationship quality and fathers’ age are provided in Table 2. As shown in that Table, many of the variables are statistically significantly correlated with each other, but with low correlation coefficients. Among those variables correlated with each other are components of men’s sexual function, such as sex drive and erectile function. Relationship quality is also associated with sexual satisfaction, with men in higher quality relationships reporting higher sexual satisfaction, as also depicted in Figure 1.

**Table 2:** **Correlations among sexuality variables, relationship quality and paternal age**

	**1**	**2**	**3**	**4**	**5**	**6**	**7**	**8**	**9**	**10**
**1 Sex drive**		.46^**^	.26^**^	.07^**^	.19^**^	.08^*^	.00	.15^**^	.03	-.01
**2 Erections**			.53^**^	.21^**^	.29^**^	-.01	.00	.05^*^	-.03	-.12^**^
**3 Ejaculations**				.22^**^	.25^**^	-.02	.01	.06^**^	-.03	-.01^**^
**4 Problem assessments**					.22^**^	-.04^*^	-.03	-.01	.01	-.12^**^
**5 Sexual satisfaction**						-.03	.02	.05^*^	-.00	-.26^**^
**6 Number of different partners**							.14^**^	.24^**^	-.13^**^	.16^**^
**7 Casual sex**								.03	-.05^*^	.08^**^
**8 Frequency of sex**									-.07^**^	.06
**9 Age**										-.02
**10 Relationship quality**										

^**^p ≤ 0.01, ^*^ ≤ 0.05.

Results of multivariate GLMs are given in Table 3. These analyses draw upon key predictors of men’s sexuality: relationship status (with the three categories of married, common-law unions, and visiting relationships employed), educational attainment, paternal age, wealth, and relationship quality. The findings show that education is the only significant predictor of sex drive, with men reporting some or completed secondary school having higher sex drive than those men with some or completed tertiary school. Men in higher quality relationships report better erectile and ejaculatory function, and fewer sexual problems. Educational attainment is also associated with ejaculatory function and problem assessments, with men having some or completed tertiary school reporting lower ejaculatory function than the preceding two educational categories and more problems compared with men with some or completed lower secondary (“Jr. High”) school.

**Table 3:** **Multivariate GLM results predicting sexuality measures**

	**Sex drive**	**Erections**	**Ejaculations**	**Problem assessment**	**Sexual satisfaction**	**No. partners**	**Casual sex**	**Frequency of sex**
**Intercept**	507.75^**^	1386.01^**^	1912.84^**^	1992.48^**^	1614.61^**^	56.97^**^	6.62^**^	9.94^**^
**Relationship status**	0.19	0.63	0.84	0.26	13.44^**^	84.19^**^	0.78	11.73^**^
**Education**	11.83^**^	6.85	17.15^**^	17.84^**^	45.61^**^	14.56^**^	8.38^*^	5.30
**Age**	2.48	0.62	0.64	0.08	0.98	16.60^**^	3.97^*^	5.77^*^
**Wealth**	1.64	0.01	0.25	0.02	5.54^*^	3.05	0.75	7.56^**^
**Relationship quality**	2.60	40.66^**^	17.34^**^	33.45^**^	197.32^**^	43.88^**^	9.71^**^	4.80^*^

Wald χ^2^ values are provided. ^**^p ≤ 0.01, ^*^ ≤ 0.05.

**Figure 2:**
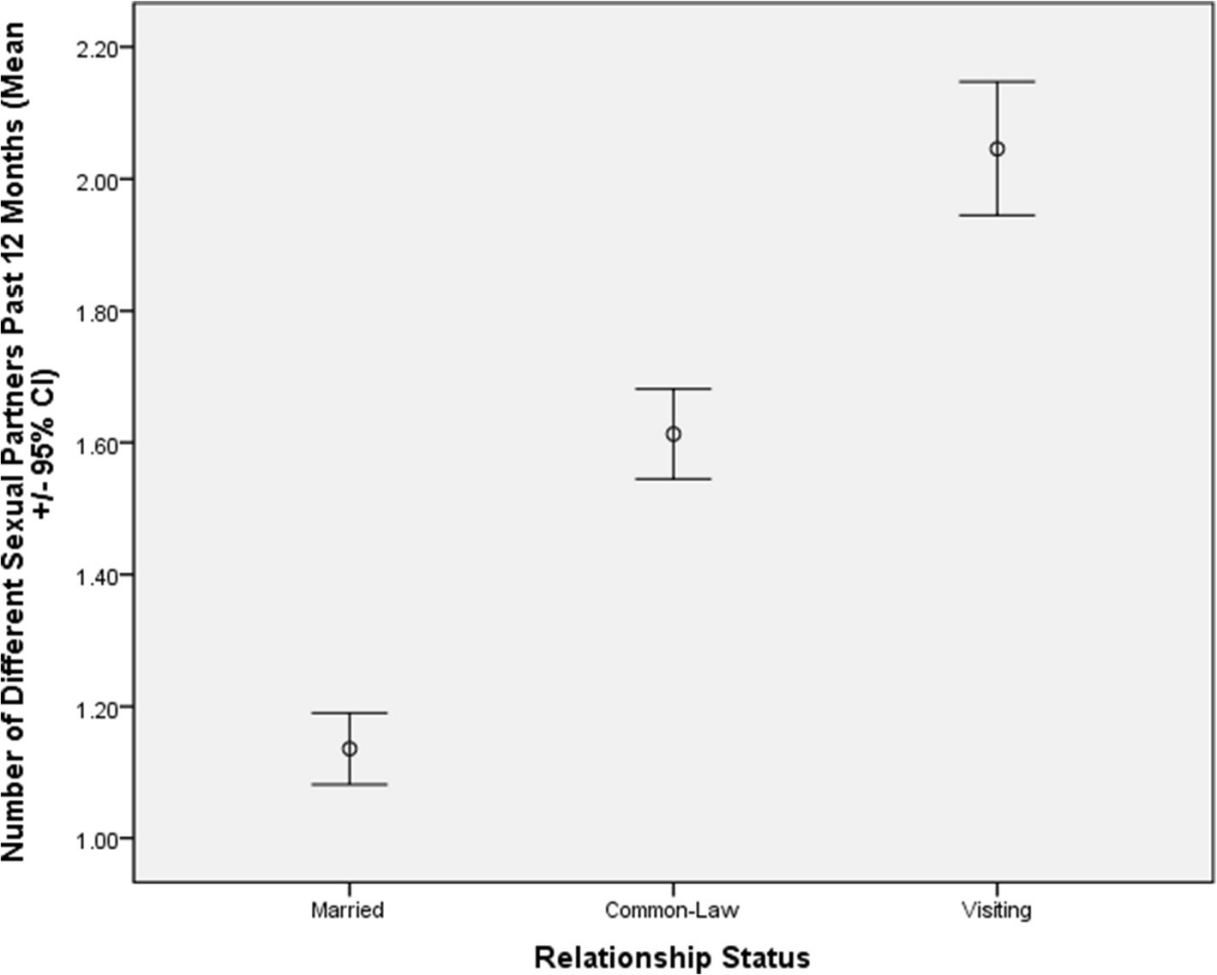
**Relationship status is associated with differences in the number of different partners reported in the past 12 months.** Married men report fewer partners than men in common-law unions, who in turn report more partners than men in visiting relationships.

Sexual satisfaction differs by four of the five predictor variables. Married men report lower sexual satisfaction than men in visiting relationships. Men in the highest category of educational attainment report lower sexual satisfaction than men in the other three categories of educational attainment. Wealth is inversely related to sexual satisfaction. And higher sexual satisfaction is related to more positive relationship quality. For number of sexual partners in the past 12 months, these vary by relationship status, with married men reporting the fewest and men in visiting relationships the most partners. These differences are also shown in Figure 2. Number of sexual partners also differs by education, with men in the highest category of educational attainment reporting fewer partners than men in the first two categories. Lower relationship quality is also associated with more sexual partners.

Openness to casual sex varies by education, with men in the highest educational category reporting less openness to sex than men in the lowest educational category. Openness to casual sex also decreases with men’s age and is inversely related to relationship quality (i.e., men in higher quality relationships are less open to casual sex). With respect to the frequency of sex in the past seven days, there were differences in relationship status. Married men reported lower frequency of sex than men in visiting relationships. The frequency of sex was negatively associated with men’s age, positively associated with wealth, positively associated with relationship quality, and also differed by education. Men reporting some or completed secondary school had higher frequency of sex than men reporting some or completed tertiary school.

## Discussion

The present study represents a large and yet relatively rare contribution to an understanding of the impacts of becoming a father on men’s sexuality. The sample size of 3410 men is unprecedented for the study of fatherhood and sexuality in the Caribbean but possibly for fatherhood and sexuality generally. That said, the sample is likely biased toward more invested partners and fathers. In order to participate, men had to report to a hospital or birth center (so were sufficiently invested to visit facilities to see a newborn and child’s mother) and were not remunerated for their efforts. After considering alternative recruitment strategies of fathers at baseline, such as phone interviews and home visits, the recruitment strategy was employed to capture many fathers in an economically efficient means. The lower percentage of visiting fathers (33%) in the present sample than previous findings by Samms-Vaughan (closer to 50%) in Jamaica is consistent with some bias toward fewer visiting fathers, who may also be in more fragile relationships with a child’s mother. We also note that the relationship dynamics of this Jamaican sample bear some similarities to the U.S. Fragile Families study, in which it has also been pointed out that visiting fathers are less likely to participate in fatherhood research than fathers in common-law or marital relationships [27]. These considerations mean that any inferences drawn from the study should recognize the potential sampling bias against less-invested fathers of newborns generally and visiting fathers more specifically.

Men’s sex drive showed more variation than did other components (erections, ejaculations, problem assessments) of sexual function. This observation suggests a fraction of men do report quite variable sex drive associated with a transition to fatherhood, adding to the literature on this topic. At the same time, as also found in previous research, men’s overall sexual satisfaction tended to be high. It could be that the relatively young age spread (e.g., mean around 29 years of age) of fathers-of-newborns in this sample contributes to the same patterns: erectile function, for example, tends to diminish at older ages than represented in the present sample, whereas men’s sex drive may be more labile at these younger ages with respect to factors such as family environment.

Approximately 30% of men reported two or more different sexual partners the previous 12 months. The time frame could include partners prior to the one with whom a man has fathered a child, meaning that this number may not align fully with concurrent partnerships or extra-pair sex during a partner’s pregnancy. This percentage is nonetheless quite high when viewed in light of inferences of multiple-partnering in Western samples. Rather than view this is an anomaly or driven primarily by reporting bias (which is always a consideration with sexuality data), there are reasons to see these data as a realistic account of partner number among fathers. These data are consistent with the wider Caribbean and Jamaican cultural contexts in which relationship fluidity is situated. An independent body of public health research on sexually transmitted infections is also consistent with ethnographic accounts of mate shifting and sexual networking, leading to the Caribbean having the highest prevalence of HIV/AIDS outside of sub-Saharan Africa [28]. A previous study on hormones and fatherhood in Jamaica we conducted also found many fathers in co-residential and visiting relationships reporting multiple sexual partners in the previous 12 months [29]. The patterning of partnering is also similar to the percentage of men in a southwest Nigerian sample of fathers who reported extramarital sex during a partner’s pregnancy, suggesting another ethnographic parallel [8].

The number of sexual partners in the past 12 months reported by fathers is also patterned in notable ways, perhaps lending further credence that these are realistic estimates. Number of sexual partners varied by relationship status, with married men reporting the fewest and men in visiting relationships the most sexual partners. These differences are consistent with the more fragile state of visiting relationships. The number of sexual partners also decreased with men’s age, varied by men’s education, and was lower among men in higher quality relationships. The age-related effect could be viewed as consistent with expectations of male reproductive senescence [30], in which males may be less sexually motivated to pursue new partners while also investing in existing offspring with advancing age. Such an age-related effect would also be consistent with age-related decreases in openness to casual sex and frequency of sexual behavior observed in the present analyses, but are at odds with the lack of age-related differences in sex drive (which would be expected to decrease with age, although this might show up with a wider age range of men than in the present sample).

Another way to view the relatively high percentage of men reporting multiple sexual partners is with respect to sexual conflicts. A growing literature highlights women’s fluctuations in sexual desire and behavior across pregnancy, postpartum and while resuming cycling. Those fluctuations can be quite profound, particularly during late pregnancy and early postpartum [3,4]. While a fraction of men report decreases in sexual desire, and we have noted variation in men’s sex drive in the present study, the magnitude of impacts of a partner’s pregnancy and in the postpartum phase on men pale in comparison to those on women. This can amplify potential sex differences in sexual desire, making these manifest at their maximum during this peripartum transition across the reproductive years [1]. The consequence is that many men may be tempted to seek additional sexual outlets if finding their sexual desires are not met with a pregnant or postpartum partner. Indeed, in their classic work on the “Human Sexual Response”, Masters and Johnson [31] suggested this could be a time in which men might stray. Hewlett and Hewlett [32] also referred to the postpartum phase as a risky time for Ngandu and to a lesser degree Aka men of Central African Republic to seek another sexual partner. Cross-culturally, societies that allow polygynous marriage (and thus alternative sexual outlets to a man) are more likely to proscribe a woman’s sexual behavior during pregnancy or postpartum [33]. A study of U.S. single parents of young children also found that fathers did not report lower dating or sexual behaviors [34]. The point of these considerations is that they may contribute to why a fraction of fathers-of-newborns are involved in multiple sexual partnerships: their sexual desires are variably but less impacted by having a child than are women’s.

At the same time, and returning to the patterning of data with respect to relationship status and relationship quality, men’s sexuality during a partner’s pregnancy and postpartum is often highly contingent upon the nature of a relationship within which a child is born and raised. In this vein, visiting relationships tend to be less and marital unions more stable, helping account for differences in sexual partner number across relationship status. A relatively high percentage of Jamaican fathers of newborns (33% in the present sample) are in visiting relationships when seen in international perspective. With the exception of sex drive, each other measure of fathers’ sexuality was associated with relationship quality. Men in higher quality relationships had fewer sexual partners, were less open to casual sex, had sex more frequently, had higher erection and ejaculation performance, fewer sexual problems, and reported more sexual satisfaction. Indeed, relationship quality tended to be the most powerful predictor of these measures of sexuality, particularly for sexual satisfaction. These findings are consistent with the idea that a high quality and stable relationship can serve as an effective channel for a man’s sexuality and paternal investment. These findings are also consistent with some larger concepts that view fathering as part of a triadic (mother-father-child) rather than dyadic (father-child) social phenomenon, and that recognize the importance of long-term partnering as a regular context in which humans tend to reproduce (unlike most other mammals) (e.g., [35]).

Of the two socioeconomic measures employed in multivariate analyses, men’s education was related to six of the eight sexuality measures, whereas men’s wealth was only related to two sexuality measures. This indicates that men’s education and wealth are not equivalent indicators of socioeconomic status in the present sample. Many of the differences with respect to education refer to more highly educated men being more likely to report less interest in sex and more problems with sex; as examples, more highly educated men reported lower erection function, interest in casual sex and sexual satisfaction. Education-related differences in men’s sexuality could represent somewhat distinct partnership strategies, with more educated men displaying a suite of characteristics indicative of more narrowly channeling their sexuality (e.g., into a single partner), but attended by lower overall sexual function. With respect to wealth, a number of cross-cultural studies have observed positive relationships between men’s status, wealth and marital partners (see [36]), and some North American studies (e.g. [37]) have found male wealth positively associated with additional access to sexual partners. However, men’s wealth in the present study was not related to number of sexual partners, though it was positively associated with frequency of recent sexual behavior. More research teasing apart the role of different measures of male socioeconomic status in relation to sexuality would be a fruitful avenue for research.

This research was subject to limitations. The data on men’s sexuality relied upon self-report. This is true of the bulk of research on human sexuality, and is a reason for interpreting the findings with caution. Findings from the study likely under-represent the prevalence of fathers in visiting relationships. This could mean that results are biased toward men in more stable and positive relationship dynamics. Women were not asked parallel sexuality questions in the cohort study, preventing the alignment of partners’ responses, even though this could be particularly informative with respect to negotiating sexuality in the face of escalating sexual conflicts during pregnancy and postpartum. More rigorous evaluation of the psychometrics of sexuality measures in a Jamaican context would be helpful. The study is of fathers of newborns, meaning that results speak largely to the impacts of a partner’s late pregnancy on men’s sexuality, but do not directly address impacts during earlier stages of a partner’s pregnancy or at specific and variable times postpartum.

## Conclusions

In summary, the present research involves an unusually large sample of men, addressing the impacts of becoming a father on men’s sexuality. Over 3000 men in Jamaica participated during a three month span in 2011. Results show that men’s sex drive is more variable than other components of sexual function such as erections or ejaculations, although overall men’s sexual satisfaction is generally high. Approximately 30% of men reported multiple partners the previous 12 months, consistent with ethnographic accounts of Caribbean relationship dynamics. Number of sexual partners was predicted by different variables, including relationship status, relationship quality and education, and can also be situated within discussions of increased sexual conflicts during the transition to childrearing. Relationship quality was regularly related to other sexuality measures, serving as the most powerful predictor of men’s sexual satisfaction. This speaks to the importance of relationship factors in structuring men’s sexuality and experience as fathers. This study thus not only provides quantitative insight into the sexuality of Jamaican fathers of newborn children, but also shows compelling patterns in men’s sexuality. The findings may also be of interest to counselors and would-be parents contemplating how a partner’s pregnancy may impact a man’s sexuality.
